# Differential Growth Rates and *In Vitro* Drug Susceptibility to Currently Used Drugs for Multiple Isolates of Naegleria fowleri

**DOI:** 10.1128/spectrum.01899-21

**Published:** 2022-02-09

**Authors:** A. Cassiopeia Russell, Dennis E. Kyle

**Affiliations:** a Center for Tropical and Emerging Global Diseases, Athens, Georgia, USA; b Department of Infectious Diseases, University of Georgia, Athens, Georgia, USA; c Department of Cellular Biology, University of Georgia, Athens, Georgia, USA; University of Illinois at Urbana Champaign

**Keywords:** *Naegleria fowleri*, drug discovery, genotype

## Abstract

The free-living amoeba Naegleria fowleri, which typically dwells within warm, freshwater environments, can opportunistically cause primary amoebic meningoencephalitis (PAM), a disease with a mortality rate of >97%. The lack of positive treatment outcomes for PAM has prompted the discovery and development of more effective therapeutics, yet most studies utilize only one or two clinical isolates. The inability to assess possible heterogenic responses to drugs among isolates from various geographical regions hinders progress in the discovery of more effective drugs. Here, we conducted drug efficacy and growth rate determinations for 11 different clinical isolates by applying a previously developed CellTiter-Glo 2.0 screening technique and flow cytometry. We found significant differences in the susceptibilities of these isolates to 7 of 8 drugs tested, all of which make up the cocktail that is recommended to physicians by the U.S. Centers for Disease Control and Prevention. We also discovered significant variances in growth rates among isolates, which draws attention to the differences among the amoeba isolates collected from different patients. Our results demonstrate the need for additional clinical isolates of various genotypes in drug assays and highlight the necessity for more targeted therapeutics with universal efficacy across N. fowleri isolates. Our data establish a needed baseline for drug susceptibility among clinical isolates and provide a segue for future combination therapy studies as well as research related to phenotypic or genetic differences that could shed light on mechanisms of action or predispositions to specific drugs.

**IMPORTANCE** Naegleria fowleri, also known as the brain-eating amoeba, is ubiquitous in warm freshwater and is an opportunistic pathogen that causes primary amoebic meningoencephalitis. Although few cases are described each year, the disease has a case fatality rate of >97%. In most laboratory studies of this organism, only one or two well-adapted lab strains are used; therefore, there is a lack of data to discern if there are major differences in potency of currently used drugs for multiple strains and genotypes of the amoeba. In this study, we found significant differences in the susceptibilities of 11 N. fowleri isolates to 7 of the 8 drugs currently used to treat the disease. The data from this study provide a baseline of drug susceptibility among clinical isolates and suggest that new drugs should be tested on a larger number of isolates in the future.

## INTRODUCTION

The amphizoic amoeba Naegleria fowleri is a protozoan eukaryote that is normally found ubiquitously in warm, freshwater environments (1). Colloquially known as the brain-eating amoeba, it is the causative agent of primary amoebic meningoencephalitis (PAM), an acute brain disease that results from amoeba-contaminated water infiltrating the nasal cavity. This allows the invasive trophozoite stage to bind to and colonize the nasal epithelium, travel through the nasal mucosa, migrate along the neuro-olfactory nerves, and traverse the cribriform plate to reach the olfactory bulbs in the central nervous system (CNS), where it feeds on neurons and damages brain membranes and meninges (1–4). Although the pathogenicity of the amoeba contributes to some of the damage, the intense immune response mounted by the host ultimately leads to death due to increased intracranial pressure and brain herniation, resulting in pulmonary edema and cardiopulmonary arrest (5). The incubation period of PAM ranges from 2 to 15 days, with >97% of cases resulting in death approximately 1 week after the initial appearance of symptoms (3, 6). This alarming mortality rate can be attributed to multiple factors, including incorrect/delayed diagnosis and ineffective clinical therapeutics. The U.S. Centers for Disease Control and Prevention (CDC) recommends a regimen of chemotherapeutics that includes amphotericin B, an azole (ketoconazole, fluconazole, posaconazole, or miconazole), azithromycin, rifampin, and miltefosine (2). More detailed guidance for health care providers regarding treatment can be found at the website provided by the CDC (https://www.cdc.gov/parasites/naegleria/treatment-hcp.html).

Out of the hundreds of cases of PAM reported by the CDC, there have only been 7 survivors worldwide with confirmed diagnoses (7). All of these survivors were treated with amphotericin B, and most also received an azole, rifampin, azithromycin, and miltefosine (7). Taravaud et al. compiled case reports in an extensive literature review to show trends in treatment selections and reported that amphotericin B and rifampicin are used most frequently, with fluconazole, azithromycin, and miltefosine trailing close behind (8). The frequent use of these drugs combined with the lack of successful outcomes leads us to speculate that there are other factors at play among amoeba populations that could contribute to treatment failure. Among these unknown factors are possible differences in growth rates, which could accelerate or slow the progression of the disease, and/or metabolism, which could potentially lead to differences in susceptibility to drugs among different amoeba isolates.

There are numerous studies that report the susceptibility of 1 to 3 clinical isolates of N. fowleri to a variety of drugs (9–29) and only 4 studies that used 5 or more different isolates (30–33). As such, we selected amphotericin B, fluconazole, ketoconazole, miconazole, posaconazole, azithromycin, rifampin, and miltefosine to assess their efficacy and determine the consistency in the response among N. fowleri clinical isolates of various genotypes, geographical locales, and temporal origins. Additionally, studies that show the growth rates of various isolates of this parasite have not been determined since the 1980s, when Haight and John showed that considerable variation of growth occurs for different strains of N. fowleri even when they are cultured in same medium under the same conditions (34). To characterize these variations in growth rates and also to rule out the possibility of varying susceptibility due to differential growth rates, we utilized flow cytometry to determine the doubling time of each of the strains. Here, we report the results of trophocidal assays using each of the aforementioned drugs, as well as the calculated growth rates for 11 clinical isolates of N. fowleri.

## RESULTS

### Comparison of drug susceptibility data among clinical isolates.

**FIG 1:**
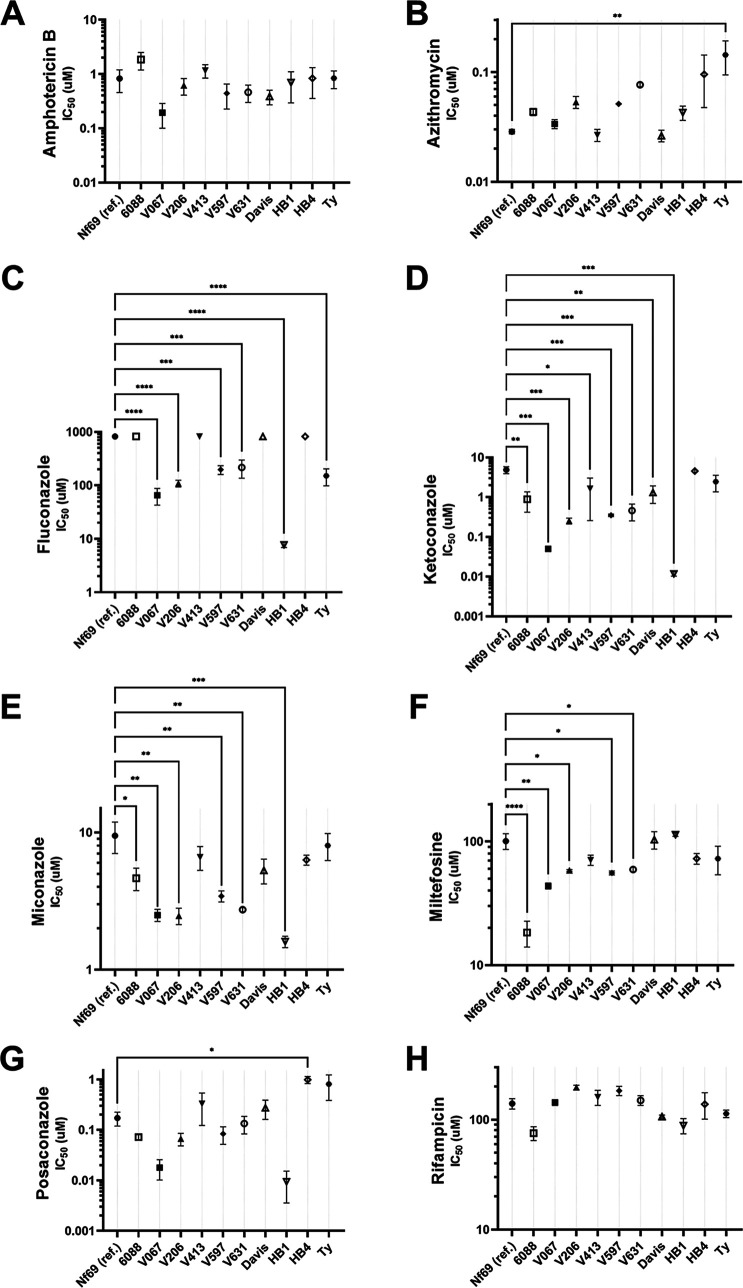
The IC_50_s of the 11 isolates determined using the CellTiter-Glo 2.0 72-h assay are shown for each of the tested drugs: amphotericin B (A), azithromycin (B), fluconazole (C), ketoconazole (D), miconazole (E), miltefosine (F), posaconazole (G), and rifampicin (H). Statistical significance was determined by one-way ANOVA with Nf69 as the reference strain. *, 0.01 < *P* < 0.05; **, 0.001 < *P* < 0.01; ***, 0.0001 < *P* < 0.001; ****, *P* < 0.0001.

We first aimed to determine the susceptibility of each clinical isolate of N. fowleri to 8 drugs commonly used and/or recommended for treatment of PAM. The 50% inhibitory concentration (IC_50_) of each drug was determined individually with three biological replicates for all 11 clinical isolates (Table 1; also, see Table S1 in the supplemental material). We used isolate Nf69 as the reference for comparisons, because it has been used extensively in recent drug discovery research (17, 23, 32, 35–38). As shown in Fig. 1A, there was no statistically significant difference between the susceptibilities of the isolates to amphotericin B and that of the reference isolate, as determined by one-way analysis of variance (ANOVA) [*F*(10,22) = 1.711; *P* = 0.1411]. For rifampicin (Fig. 1H), although the one-way ANOVA results indicated that there was significant variance among the set of isolates as a whole, *post hoc* Dunnett’s multiple-comparison tests showed no statistically significant differences between Nf69 and the other 10 isolates [*F*(10,22) = 4.618, *P* = 0.0013]. For azithromycin (Fig. 1B), the IC_50_ for strain TY was more than four times higher than that for Nf69, 0.14 ± 0.05 μM versus 0.029 ± 0.001 μM, respectively. This difference was determined to be statistically significant with one-way ANOVA [*F*(10, 22) = 2.944, *P* = 0.0166] and *post hoc* Dunnett’s multiple-comparison test (*P* = 0.0066). When evaluating the effectiveness of fluconazole (Fig. 1C), we utilized an upper cutoff of 820 μM, and 5 of the 11 isolates (including the reference) surpassed this cutoff, showing a lack of inhibition by this compound in nearly half of the clinical isolates tested. Furthermore, the IC_50_s for the remaining isolates were found to be significantly lower than that for Nf69, which was reported as 820 μM due to a lack of inhibition even at the highest concentration of fluconazole tested [*F*(10,12) = 34.91, *P* < 0.0001]. For V067, V206, V597, V631, HB1, and TY, the IC_50_s of fluconazole were 65.3 ± 22.7 μM, 109 ± 16.0 μM, 196.7 ± 36.7 μM, 216.7 ± 80.9 μM, 7.73 ± 0.87 μM, and 150.7 ± 52.3 μM, respectively.

**TABLE 1:** Clinical isolate patient/regional information, growth rates, genotypes and literature references

Isolate (alternative identifier)	Patient sex/location/yr	Mean growth rate (h) ± SEM^*a*^	Genotype^*b*^	Reference(s)
Nf69 (ATCC 30215)	M/Australia/1969	20.3 ± 2.1	IV^*c*^/5^*c*^	17, 23, 28, 32, 44, 46–49
V067 (V523)	M/Arizona/1987	21.4 ± 1.0	III/3^*c*^	32, 39, 40, 52
V206	M/Mexico/1990	24.4 ± 0.2	I/2^*c*^	32, 39, 40
V413	M/Texas/1998	18.5 ± 0.5	I/2^*c*^	32, 39, 40, 52
V597	M/Florida/2007	29.6 ± 0.7	I/2^*c*^	40
V631	M/Louisiana/2011	24.6 ± 1.0	I/2^*c*^	40, 53
6088 (ATCC 30896; CAMP)	F/California/1978	23.7 ± 0.6	II/1^*c*^	40, 50, 51
Davis (V414)	M/Florida/1998	22.4 ± 0.7	I/2^*c*^	31, 40
HB1 (Hb)	M/Florida/1966	19.0 ± 1.2	III/3^*c*^	12, 19, 25, 26, 40, 46, 54–57
HB4	F/Virginia/1977	21.7 ± 1.2	III/3^*c*^	40, 51, 57, 58
TY	M/Virginia/1969	22.9 ± 1.9	III/3^*c*^	31, 33, 40, 43, 51, 59

a Mean growth rates were determined in this study.

b Genotyping is reported according to 2 methods, in this format: Roman numeral (according to Zhou et al. [39])/Arabic numeral (according to De Jonckheere [41]).

c Genotype determination made in this study.

Eight of the clinical isolates showed significantly increased susceptibility to ketoconazole compared to Nf69, which had an IC_50_ of 4.87 ± 0.97 μM [*F*(10,22) = 6.913, *P* < 0.0001]. The IC_50_s were 0.89 ± 0.47 μM, 0.05 ± 0.006 μM, 0.25 ± 0.043 μM, 1.64 ± 1.38 μM, 0.35 ± 0.026 μM, 0.46 ± 0.21 μM, 1.31 ± 0.62 μM, and 0.012 ± 0.001 μM for 6088, V067, V206, V413, V597, V631, Davis, and HB1, respectively. For miconazole (Fig. 1E), 6 of the isolates were significantly more susceptible than Nf69 [*F*(10,22) = 5.338, *P* = 0.0005], with IC_50_s of 9.48 ± 2.4 μM, 4.63 ± 0.86 μM, 2.5 ± 0.34 μM, 2.47 ± 0.34 μM, 3.43 ± 0.32 μM, 2.73 ± 0.088 μM, and 1.6 ± 0.15 μM for Nf69, 6088, V067, V206, V597, V631, and HB1, respectively. Five isolates showed increased susceptibility to miltefosine compared to the value of 100.7 ± 14.6 μM for Nf69 [*F*(10,22) = 8.64, *P* < 0.0001], with IC_50_s of 18.3 ± 4.3 μM, 43.7 ± 2.4 μM, 58.3 ± 0.88 μM, 55.7 ± 2.0 μM, and 59.3 ± 2.3 μM for 6088, V067, V206, V597 and V631, respectively. Last, HB4 was the only isolate to show a significant decrease in susceptibility to posaconazole, with an IC_50_ of 0.98 ± 0.16 μM (Fig. 1G), which is more than 4 times higher than that of 0.17 ± 0.052 μM for Nf69 [*F*(10,22) = 4.449, *P* = 0.0017]. It is possible that sulfisoxazole, one of the drugs administered to the surviving patient in 1978 (6088), could have contributed to this positive outcome, so we also tested its efficacy on each of the isolates. Although this compound was tested at concentrations up to 940 μM, it was not found to affect the growth of any of the isolates compared to controls (data not shown). Additional IC_50_s (with standard errors of the means [SEM]) can be found in Table S1.

### Growth rate results.

By utilizing the absolute count feature of FCS Express software when analyzing our flow cytometry data, we were able to develop a semiautomated counting technique for amoebae that excluded debris, which is common when culturing free-living amoebae; this method was used to monitor the growth rates of the 11 clinical isolates included in this study. The generation time of these amoebae ranged from 18.5 ± 0.5 h for V413 to 29.6 ± 0.7 h for V597 (Table 1), which is about 1.5 times slower than that of the fastest-growing isolate. The reference isolate, Nf69, was one of the fastest-growing isolates, with a generation time of 20.3 ± 2.1 h. The overall average growth rate of the clinical isolates was found to be 22.4 ± 0.9 h. We found multiple statistically significant differences among the isolates as determined by one-way ANOVA [*F*(10,22) = 7.184, *P* < 0.0001]. *Post hoc* Tukey-Kramer’s multiple-comparison test showed significant differences between the generation times of Nf69 versus V597 (*P* = 0.0004), V067 versus V597 (*P* = 0.0019), V206 versus V413 (*P* = 0.0445), V413 versus V597 (*P* < 0.0001), V413 versus V631 (*P* = 0.0343), V597 versus 6088 (*P* = 0.0445), V597 versus Davis (*P* = 0.0076), V597 versus HB1 (*P* < 0.0001), V597 versus HB4 (*P* = 0.0028), and V597 versus TY (*P* = 0.0152) (Table S2). V597, which was the slowest-growing strain, accounted for 8 of the 10 differences in generation times, while V413, the fastest-growing strain, accounted for the other 2 differences. To confirm that the growth rate was not a confounding factor when comparing susceptibility of drugs among isolates, we performed a bivariate analysis of the growth rates versus the IC_50_s (Fig. S3) and found that there was no correlation between the susceptibility of the amoebae to the drugs and the speed of their growth.

### Nf69 genotyping results.

In addition, we wanted to compare the drug susceptibilities of the clinical isolates with their genotypes. There is published internal transcribed spacer (ITS) genotype information, determined based upon the techniques described by Zhou et al., for all of the isolates except Nf69 (39, 40). After obtaining Sanger sequencing results for the PCR products for Nf69, we trimmed off poor-quality base pairs and aligned the sequences obtained for the forward primer sequencing samples as well as the reverse primer sequencing samples to attain a 446-bp sequence. We identified the forward primer and the complement of the reverse primer within the sequence and then followed the recommendation to make a genotype determination (41). We identified 1 copy of repeat 2 (ATGGTAAAAAAGGTGAAAACCTTTTTTT), 1 copy of main 2 (ATGGTAAAAAAGGTGAAAACCTTTTTT), and 1 copy of main 1 (CCATTTACAAAAAAT). To make the final identification, we found repeat 1 (ATGGTAAAAAAGGTGT) to have a 2-bp deletion of the A and G residues at the 11th and 12th positions, respectively, and also a C nucleotide at position 31 in the 5.8S rDNA that followed the 84-bp ITS1 sequence. With all of these findings, we were able to conclude that Nf69 falls within genotype 5, previously described in the literature for the species N. fowleri (41, 42). We also followed the less detailed protocol described by Zhou et al. and found that Nf69 falls within genotype IV (39).

A recent study by Joseph et al. sequenced the genomes for each of the isolates tested in our study, and we downloaded and processed these data in order to perform the aforementioned genotyping technique and compare the genotype results attained with the system created by De Jonckheere with those reported based on the work of Zhou et al. (39–41). Using Geneious Prime software, we pairwise aligned the forward and reverse reads for each clinical isolate and then mapped these to each of the 2 ITS primers. We then *de novo* assembled these reads, which resulted in 2 contigs per isolate, one which bound the forward primer and the other the reverse primer. We extracted and aligned sequences from each contig to obtain a consensus sequence that was then processed to determine the genotypes (see Materials and Methods for a more detailed description). We found that the isolates defined at genotype I by the system of Zhou et al. are defined as genotype 2 by De Jonckheere’s methods, and those that are genotype II are genotype 1, respectively (Table 1) (39, 41). Additionally, genotype III according to Zhou et al. was equivalent to genotype 3 by De Jonckheere’s definition, and genotype IV was equivalent to genotype 5.

## DISCUSSION

This is the first study showing the *in vitro* susceptibilities of more than 10 clinical isolates of Naegleria fowleri to the currently recommended chemotherapeutics for the devastating neurological infection PAM. In the literature, *in vitro* drug susceptibility testing for PAM has progressed incrementally since the disease was first discovered in the late 1960s. Initial assays developed for amoebae viability to chemotherapeutics all involved manual counting and potentially subjective phenotypic determination. In 1975, Schuster and Rechthand observed the amoebicidal as well as the ultrastructural effects of amphotericin B with *in vitro* testing of the nonpathogenic species Naegleria gruberi and the Carter and TY strains of N. fowleri (43). Although they did not explicitly report an IC_50_, they showed amphotericin B to be amoebicidal at concentrations of 0.25 μg/ml (0.271 μM) and greater for TY, an observation that we closely reproduced in our assay with an IC_50_ of 0.73 ± 0.3 μM (Table S1) (43). In 1976, Duma and Finley performed a study with 6 different clinical isolates of N. fowleri and tested amphotericin B, clotrimazole, and miconazole *in vitro* against all of the organisms (33). Of the clinical isolates tested, TY is the only one that we also utilized in our study, and upon comparing our data for amphotericin B with their MIC of 0.422 μM, we found that it also overlaps nicely with our IC_50_ of 0.73 ± 0.3 μM (Table S1). Additionally, they reported a MIC of 2.98 μM for miconazole against TY, which is similar to the IC_50_ of 7.6 ± 1.8 μM in this study (33).

Subsequent studies in the late 1970s and early 1980s all reverted to the use of a single clinical isolate to determine chemotherapeutic susceptibility *in vitro* (9–13). The practice of utilizing multiple isolates to reconfirm susceptibilities to novel therapeutics was not revisited until 2006, when Schuster et al. utilized 3 different strains to perform *in vitro* testing of miltefosine and voriconazole (27). There was no overlap in the isolates used for this study and ours; however, miltefosine was used, and the authors showed that a minimum of 55 μM was required to attain amoebicidal conditions, which falls within our range of IC_90_s from 21.6 μM to >120 μM shown across isolates (Table S1). Importantly, these authors also observed strain variations in sensitivity to the drugs they tested on amoebae (27). Further technical advances were made by Kim et al. in 2008 with the optimization of a colorimetric lactate dehydrogenase release assay to measure amoebicidal activity of compounds *in vitro* (28). This provided the groundwork for the development and optimization of high-throughput screening assays to identify novel chemotherapeutics to treat PAM (16, 17).

Surprisingly, even with the establishment of high-throughput assays for testing compounds against this amoeba, it took another decade for the inclusion of more than a single isolate of N. fowleri in drug testing studies. In 2018, Peroutka-Bigus et al. used 2 different isolates, LEE and HB1, to determine amoeba viability against auranofin. The authors noted a significant difference in sensitivity between the 2 amoebae and indicated that clinical outcomes could be dependent on individual strain susceptibilities to drugs (19). In 2019, Colon et al. (32) utilized a high-throughput screening method to identify novel drugs to treat PAM using 6 different isolates: Nf69, V067, V206, V413, V414, and V596. They compared the *in vitro* drug susceptibilities to 4 chemotherapeutics and showed no statistical difference between Nf69 and the other 5 isolates for amphotericin B, azithromycin, miltefosine, and posaconazole (32). Our study, which incorporated 4 of the 6 clinical isolates used by Colon et al., reiterates the lack of significant differences between isolates for amphotericin B as well as azithromycin and posaconazole. However, due to our inclusion of additional strains, we were able to detect variance in two of the isolates not tested by Colon et al.—TY for azithromycin and HB4 for posaconazole—which further emphasizes the need for multi-isolate drug testing in N. fowleri. To further showcase the recent trend in the right direction, in 2020, Escrig et al. incorporated 5 different clinical isolates of various genotypes and geographic origins to showcase the variance in susceptibility to the tested chemical compounds (31). Their average 50% effective concentration (EC_50_) of 37.8 μM for miltefosine falls within the range of IC_50_s obtained in our study for the same drug. With an average EC_50_ of 0.094 μM for amphotericin B, their data indicated a more potent response to this molecule than ours, but this could be due to differences in assay parameters (e.g., 10,000 versus 4,000 amoebae/well and a 48-h versus a 72-h time point), or potential batch-to-batch variation in molecule potency.

Furthermore, we report the sequence of a portion of the small-subunit (SSU) rRNA gene, ITS1, the 5.8S rRNA gene, ITS2, as well as a portion of the LSU RNA gene of Nf69 that we attained in order to determine the genotype of this isolate. We initially endeavored to ascertain the genotyping parameters by referring to the resource used by Ali et al. in their recent publication, in which they defined the TY isolate as genotype III (44). This guideline, provided by Zhou et al., which uses Roman numerals and defines genotypes I to VI (39), is outdated, and the repeats and various components of the ITS1 as well as 5.8S rRNA gene are less detailed than in the more recent review by De Jonckheere, which uses Arabic numerals to differentiate types based upon the evolutionary consideration of which type likely appeared first (41). As such, there is a need for the establishment of a universal set of genotyping parameters to maintain consistency among publications and future research when characterizing isolates of N. fowleri.

Not only have we shown statistically significant variability in IC_50_s among the clinical isolates that were tested, but we also calculated significant differences in growth rates by culturing each isolate under controlled equivalent conditions. Previous research by Weik and John showed that agitated cultures allow a speedy generation rate of approximately 5.5 h with higher maximum yields of amoebae than unagitated amoebae (45). This difference in generation time is likely attributable to the higher incubation temperature of 37°C— compared to our selection of 34°C to maintain consistency with previous studies published by our lab (17, 23, 32, 35)—and also to the maximum population density of the small volume of medium used in this study. We speculate that our calculated doubling rates would likely be lower with higher temperatures or increased volume/allotted growth area.

Upon diagnosis, the time frame for treatment is already highly compacted due to the fulminant nature of PAM, and the use of drugs that the amoebae have various levels of innate resistance to is potentially detrimental. These data provide valuable evidence that the universal approach of applying the recommended cocktail of drugs might not be the most effective one. Overall, a lack of variety in genotypic as well as geographic origins could lead to the premature conclusion that a newly discovered compound or scaffold is universally effective against N. fowleri when there could be a notable difference in activity across different isolates of the parasite. Thus, the findings in this paper draw attention to the changes that need to be made in the field in treating and discovering new therapeutics for this deadly disease. Moving forward, multiple isolates of various genotypes should be used when the susceptibility of this parasite to prospective drugs and bioactive molecules is determined. This has long been the standard of practice for antimicrobial drugs, and our data support the need for this change to be adopted for drug discovery for N. fowleri.

The scientific and medical community also needs to reevaluate the effectiveness of the currently recommended therapeutics for treating PAM. It is important to recognize that the most commonly used drug regimen(s) were derived empirically and based upon the few successful treatment regimens. More recently, repurposed drugs (e.g., miltefosine) have emerged from laboratory studies, yet the entire drug cocktail used to treat PAM does not have sufficient *in vitro* or *in vivo* (mouse model) data. For example, we have shown that fluconazole and rifampicin are not potent for the majority of the 11 isolates tested, and thus, continued use of these two drugs in the recommended treatment regimen should be reconsidered. With regard to azoles, we recommend treating patients with posaconazole rather than the less potent azoles fluconazole, miconazole, and ketoconazole.

The overarching need for this disease is a more targeted set of recommendations according to the genotype, growth rate, and susceptibility of the specific isolate, and the components of the therapeutic cocktail should be targeted to these important elements in order to give patients a higher chance for surviving an infection by N. fowleri. The empirical results reported herein should be considered in light of our reported efficacy of single-entity therapy versus the standard combinational therapy for PAM. Future studies that determine combinational effects of the different drugs, whether they be synergistic or antagonistic to each other, should be performed on multiple isolates. Performing these studies with a combination of 3 or more drugs might be challenging but is warranted due to the acute nature of this infection and the urgent need for more effective treatment options.

### Conclusion.

In conclusion, we determined that there is a statistically significant difference in the susceptibility to the majority of the currently recommended drugs for PAM among clinical isolates of N. fowleri. These data show that the current therapeutic recommendations should be re-examined and help to establish a baseline for drug susceptibility among different clinical isolates. It also paves the way for the identification of differences between isolates, whether they are genetic elements, such as single nucleotide polymorphisms (SNPs) or other mutations, or phenotypic elements that may provide a hint as to a predisposition to specific drugs. Regardless of the approach, we show that a more targeted methodology is needed in order to focus on the specific amoebae infecting a patient and subsequently increase the odds for survival.

## MATERIALS AND METHODS

### Naegleria fowleri clinical isolates.

Nf69 (ATCC 30215), a clinical isolate used as a reference strain in these studies and obtained from a 9-year-old boy in Adelaide, Australia, who died in 1969 (46–49), and 6088 (ATCC 30896), obtained from a 9-year-old girl in California who survived in 1978 (50, 51), were purchased from the American Type Culture Collection (ATCC). V067, isolated from a 30-year-old male in Arizona who died in 1987 (39, 52), V206, isolated from a man in Mexico who died in 1990 (39), V413, isolated from a 17-year-old boy in Texas who died in 1998 (39, 52), V597, isolated from a 10-year-old boy in Florida who died in 2007, V631, isolated from a 28-year-old man in in Louisiana who died in 2011 (53), Davis, isolated from an individual in Florida who died in 1998 (31), HB1, isolated from a 16-year-old boy in Florida who died in 1966 (25, 54–57), HB4, isolated from a female in Virginia who died in 1977 (51, 58, 59), and TY, obtained from a 14-year-old boy in Virginia who died in 1969 (31, 33, 44, 51, 60), were all kindly provided by Ibne Ali at the Centers for Disease Control and Prevention (CDC).

### Culturing of amoebae.

Trophozoites were grown axenically at 34°C and 5% CO_2_ in nonvented 75-cm^2^ tissue culture flasks (Olympus, El Cajon, CA, USA) with Nelson’s complete medium (NCM) supplemented with 10% fetal bovine serum (FBS) and 100 U/ml penicillin and 100 mg/ml streptomycin until 80 to 90% confluent. For subculturing, flasks were placed on ice for approximately 15 min to detach adherent cells, and cells were collected via centrifugation at 4°C for 5 min at 4,000 rpm. The resulting amoeba pellet was then resuspended in 1 ml of NCM supplemented with 10% FBS and either passaged to new flasks or diluted further for manual counting with a hemocytometer. These counts were performed in duplicate, and the resulting mean was used to calculate the dilutions needed for microwell plates (4,000 cells per well) and microcentrifuge tubes (35,000 cells per tube).

### Drug sources and preparations.

Amphotericin B (CAS number 1397-89-3), azithromycin (CAS number 117772-70-0), ketoconazole (CAS number 65277-42-1), and posaconazole (CAS number 171228-49-2) were purchased from Sigma-Aldrich (St. Louis, MO). Miconazole (CAS number 22916-47-8) was purchased from Millipore Sigma (Sigma-Aldrich, St. Louis, MO). Miltefosine (CAS number 58066-85-6) was purchased from Cayman Chemical (Ann Arbor, MI), rifampicin (CAS number 13292-46-1) was obtained from Fischer Scientific (Hampton, NH), and fluconazole (CAS number 86386-73-4) was purchased from the United States Pharmacopeia (Rockville, MD). Working stocks for all compounds were created by dissolving the drugs in dimethyl sulfoxide (DMSO) at 5-mg/ml concentrations with the exception of fluconazole, which was prepared at 25 mg/ml.

### CellTiter-Glo 2.0 *in vitro* drug susceptibility assay.

We used the CellTiter-Glo 2.0 reagent kit (CTG; Promega, Madison, WI) to determine the 50% IC_50_s of each drug for amoeba isolates, as previously described (17, 23, 32, 35). To identify the optimal concentration of cells to seed for this assay and also to confirm that this falls within the linear range of the luminescence-based assay, the optimal seeding density of each of the isolates was determined by seeding a dilution series of trophozoites (ranging from 1,000 cells to 10,000 cells) in Thermo Scientific Nunc flat-bottomed 96-well microwell white polystyrene plates (Thermo Fisher Scientific, Waltham, MA). Drug susceptibility assays involved serial dilutions of each drug initially from 50 μg/ml to 5 ng/ml (250 μg/ml to 25 ng/ml for fluconazole and starting at lower concentrations as needed for more potent drugs) with 4,000 amoebae/well and a total volume of 100 μl/well to generate dose-response curves. Control wells were cultured in triplicate per plate with 1% DMSO for the negative controls and 54.1 μM amphotericin B for the positive controls. Each isolate was normalized to its own controls due to variability in average luminescence produced among isolates. Plates were incubated at 34°C and 5% CO_2_ for 72 h.

At the 72-h time point, 25 μl of CellTiter-Glo 2.0 reagent was added to each well. Plates were protected from light, and well contents were mixed to facilitate cell lysis with an orbital shaker at 300 rpm for 2 min at room temperature. Plates were equilibrated for 10 min, per the manufacturer’s recommendation, to stabilize the luminescent signal prior to measurement. The luminescent signal, reported as relative light units (RLU), is directly proportional to the amount of ATP in each well and was measured at 490 nm with a SpectraMax I3X plate reader (Molecular Devices, Sunnyvale, CA, USA). To generate drug susceptibility curves, ATP RLU curve fitting was performed with Collaborative Drug Discovery, Inc., software (CDD Vault, Burlingame, CA, USA). To measure percent inhibition, negative controls were calculated using 1% DMSO and positive controls were calculated with 54.1 μM amphotericin B, and these controls were applied for each isolate within each plate to normalize the data. Nonlinear regressions were performed using the Levenberg-Marquardt algorithm within CDD Vault. IC_50_s were obtained from 3 biological replicates, each consisting of 2 technical replicates per drug concentration, and with the standard error of the mean (SEM). Dose-response graphs were prepared by using GraphPad Prism software version 9.0 (GraphPad, La Jolla, CA, USA).

### Growth rate calculations via flow cytometry.

With the goal of obtaining absolute counts of the amoebae in an easily reproducible manner, we seeded 35,000 amoebae in 1.5 ml of NCM supplemented with 10% FBS, 100 U/ml penicillin, and 100 mg/ml streptomycin in 1.5-ml microcentrifuge tubes and incubated these at 34°C and 5% CO_2_ for 72 h. At the 72-h time point, tubes were centrifuged at 14,000 rpm for 5 min at room temperature, medium was carefully aspirated from pellets, and 250 μl 1× phosphate-buffered saline (PBS) was added to each tube. To fix the amoebae, 250 μl of 4% paraformaldehyde (PFA) was added, and the pellets were resuspended via gentle pipetting. Cells were incubated in the resulting 2% PFA mixture for 15 min at room temperature before being centrifuged at 14,000 rpm for 5 min, followed by careful removal of the supernatant and resuspension of the pellet in 0.5 ml of 1× PBS. No viability dye was added, as we wanted to limit the amount of manipulation to prevent possible loss of cells and attain more accurate counts of amoebae. An additional manual count of each of the isolates was performed in duplicate for one of the 3 biological replicates following the 72-h incubation within microcentrifuge tubes; these data reconfirmed the accuracy of flow cytometry gating and counts (data not shown).

All samples were analyzed using a NovoCyte Quanteon flow cytometer (ACEA Biosciences, San Diego, CA, USA) to perform an absolute count based upon light scatter. The strategy used for gating cells (Fig. S2) involved exclusion of debris in the reference sample (Nf69) as well as inclusion of the majority of events in order to account for differences in size among amoebae populations. This gating was then applied to the remaining samples to maintain consistency between isolates. These data were analyzed using FCS Express software version 7.06.0015 (De Novo Software, Pasadena, CA).

To calculate the generation time of each of the clinical isolates, the following equation for bacterial growth by binary fission was adapted to suit our needs.

(1) $$G=\frac{t}{n}$$

The generation time, *G*, is equal to time in hours, *t*, divided by number of generations, *n*, with *n* being the number of times the cell population doubles during the specified time interval, calculated with the following equation:

(2) $$a=A\times 2^{n}$$

The number of amoebae at the end of the time interval was defined as *a*, and the number of amoebae at the beginning of a time interval was defined as *A.* Simplification of equation 2 to solve for *n* and substitution into equation 1 provided the final equation (equation 3), which was utilized for the reported generation time calculations:

(3) G=t3.3 log⁡aA

### Genotype determination and method comparison.

Because the genotype for Nf69 has not been previously reported in the literature, we used the techniques described by De Jonckheere (41) to determine the genotype for this isolate. Genomic DNA extractions were performed upon 5 million trophozoites of Nf69 using a Quick-DNA Miniprep Plus kit according to manufacturer recommendations (Zymo Research, Irvine, CA, USA). In short, we utilized the following ITS primers for *Naegleria* sp.: ITSFWD (5′-AACCTGCGTAGGGATCATTT-3′) and ITSREV (5′-TTTCCTCCCCTTATTAATAT-3′). The PCR amplification was carried out with Phusion high-fidelity PCR master mix with HF buffer in 50-μl reaction mixtures with 20 ng of genomic DNA (New England Biolabs Inc., Ipswich, MA, USA). Reactions were run in an Agilent SureCycler 8800 (Agilent Technologies, Santa Clara, CA, USA) for 6 min at 94°C to allow DNA denaturation and then for 35 cycles of 94°C for 1 min, 55°C for 1 min and 30 s, and 72°C for 2 min with a final elongation step for 10 min at 72°C. Following the PCR cycling, 12 μl of the PCR was mixed with 2 μl of 6× DNA loading dye (Thermo Fisher Scientific, Waltham, MA, USA), and this was run in a 1.5% agarose gel at 125 V for ∼90 min. The GeneRuler 1 kb Plus DNA ladder (Thermo Fisher Scientific, Waltham, MA, USA) was used as a size marker, and the band of ∼400 bp was excised and purified using the MinElute gel extraction kit according to the manufacturer’s protocols (Qiagen, Hilden, Germany). The resulting purified PCR products were sent for Sanger sequencing in both the forward and reverse directions by Genewiz sequencing services (South Plainfield, NJ, USA). Poor-quality bases were trimmed from either end using Geneious Prime software version 2020.2.5 (Biomatters Ltd., Auckland, New Zealand), and the consensus between the forward and reverse sequences as well as duplicate samples was extracted for further analyses.

To compare genotype determinations for each of the two methods that are reported in the literature (39, 41), we accessed the genome sequences recently published under BioProject no. PRJNA642022 and imported them into Geneious Prime software as paired reads in order to obtain pairwise alignments (options: Illumina, paired end [inward pointing]; pairs of files, 500 bp) for each clinical isolate included in this study (40). We then mapped the reads to the ITSFWD and ITSREV primers using the Geneious mapper with medium-low sensitivity, allowing iterations up to 5 times for fine tuning (all other options were set to default). The results of this mapping step were then *de novo* assembled to each other with the Geneious assembler (with medium sensitivity and default parameters; the option to dissolve the contigs and reassemble was not selected), which resulted in 2 contigs—one that bound the ITSFWD primer and one that bound the ITSREV primer. The overlapping consensus sequences from the FWD contig as well as the reverse complement of the REV contig were extracted, and genotyping was performed and reported in Table 1, as described in Results (41).

### Statistical analyses.

We used the Z′ factor as a statistical measurement to confirm the validity of the drug susceptibility screening assay. By considering the means and standard deviations for the positive and negative controls of each drug plate, Z′ assesses data quality and robustness of each plate to indicate the probability of false positives or negatives. All of the plates screened had excellent Z′ scores, >0.56.

To determine if there were statistically significant differences in IC_50_ for each drug and growth rates among the isolates, one-way ANOVA with Dunnett’s multiple-comparison and Tukey-Kramer’s multiple-comparison *post hoc* tests, respectively, were performed using GraphPad Prism version 9.1.2 (GraphPad, La Jolla, CA, USA).

### Data availability.

The Naegleria fowleri Nf69 446 bp sequence containing a portion of the SSU rRNA, ITS1, 5.8S rRNA, ITS2, and a portion of the LSU rRNA has been deposited in GenBank under accession number MZ494674.
