# Supplementary material for: Differential Growth Rates and In Vitro Drug Susceptibility to Currently Used Drugs for Multiple Isolates of Naegleria fowleri
Source: Microbiol Spectr. 2022 Feb 9;10(1):e01899-21. doi: 10.1128/spectrum.01899-21 (PMC8826828; doi:10.1128/spectrum.01899-21)
Supplement: SUPPLEMENTAL FILE 1 — Supplemental material. Download SPECTRUM01899-21_Supp_1_seq1.pdf, PDF file, 0.6 MB [file spectrum01899-21_supp_1_seq1.pdf]

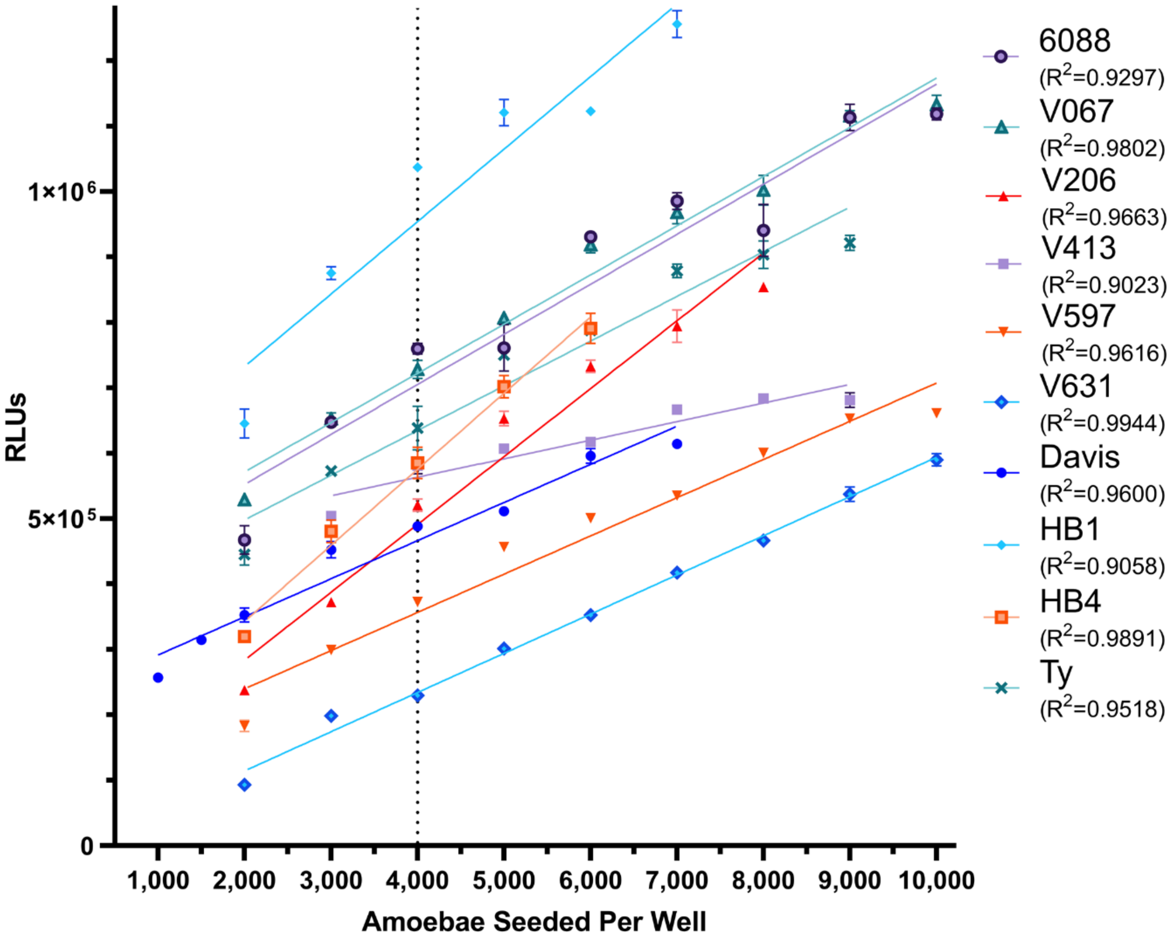

FIG S1 Optimal seeding density for the CellTiter Glo 2.0 kit determined by linear regression with at least 5 serial dilutions per clinical isolate. Three technical replicates were performed per concentration per clinical isolate. The optimal seeding density for *Nf69* was previously determined to be 4000 amoebae per well (1).

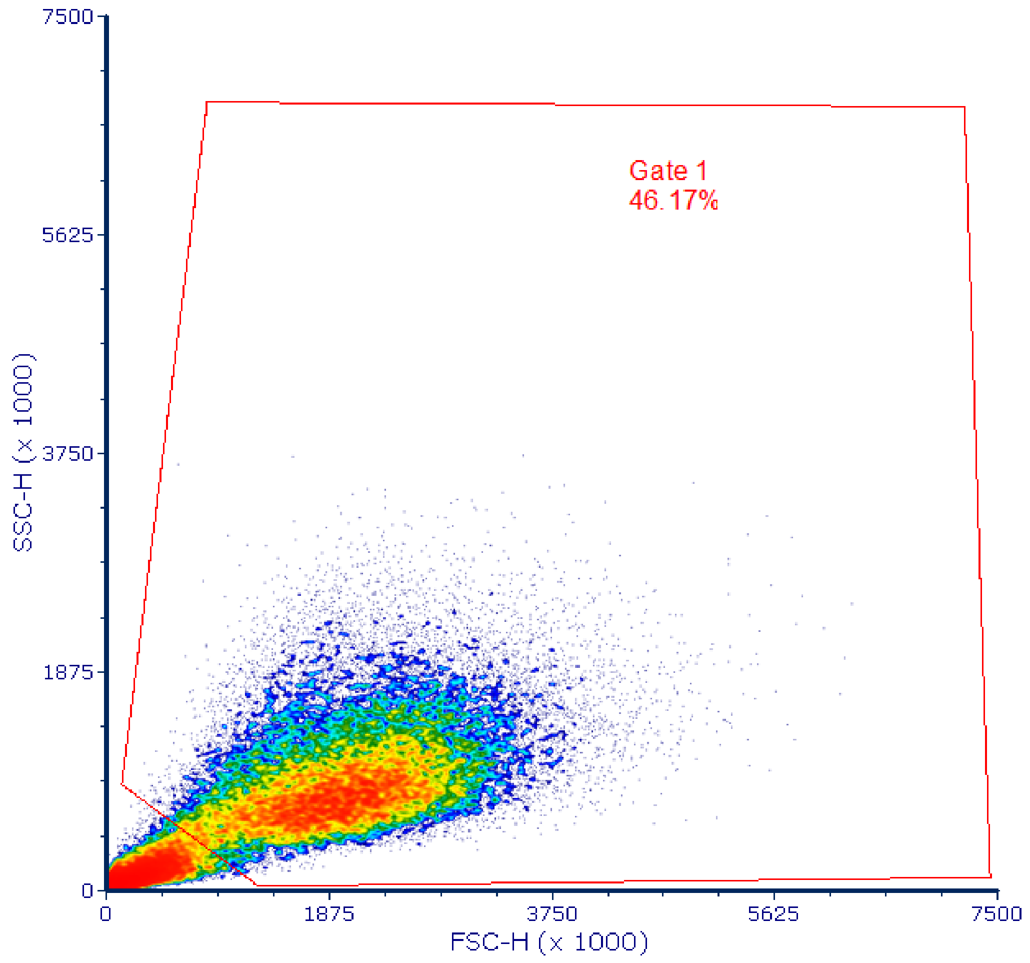

FIG S2 Representative gating strategy in a reference sample (*Nf69*) utilized for the forward-scatter (FSC-H) versus side-scatter (SSC-H) results attained for all clinical isolates. This gating strategy was applied to all samples in order to maintain consistency in the absolute counting of amoebae.

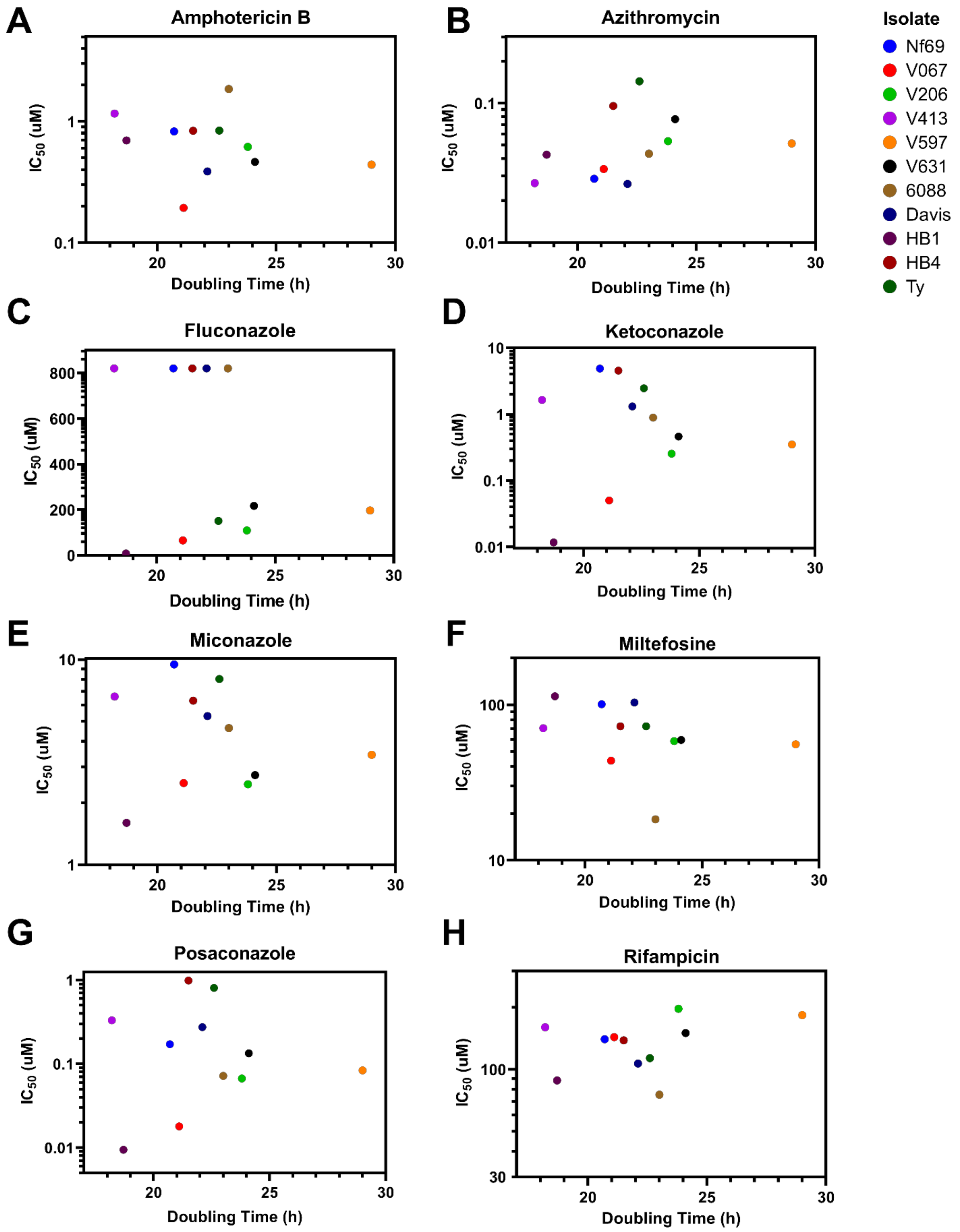

FIG S3 Bivariate analysis of doubling time vs IC50 for each of the drugs and isolates. These data suggest there is no correlation between doubling time and susceptibility to any of the drugs tested.

TABLE S1 IC50/90s (µM) ± the standard error of the mean determined for 11 clinical isolates of *N. fowleri.* We used 3 biological replicates, each with 2 technical replicates per concentration tested to generate dose response data.

Clinical Isolate		Chemotherapeutic							
		Amphotericin B	Azithromycin	Fluconazole	Ketoconazole	Miconazole	Miltefosine	Posaconazole	Rifampicin
Nf69	IC_{50} ± SEM	0.63 ± 0.37	0.026 ± 0.001	> 820*	4.69 ± 0.97	8.84 ± 2.45	98.3 ± 14.6	0.16 ± 0.05	138.4 ± 15.3
	IC_{90} ± SEM	0.69 ± 0.39	0.028 ± 0.000	> 820*	10.3 ± 1.52	12.7 ± 2.05	116.0 ± 12.3	0.36 ± 0.08	>300*
6088	IC_{50} ± SEM	1.61 ± 0.66	0.04 ± 0.003	> 820*	0.64 ± 0.47	4.48 ± 0.87	18.6 ± 3.93	0.07 ± 0.01	73.9 ± 10.9
	IC_{90} ± SEM	1.73 ± 0.74	0.42 ± 0.25	> 820*	2.90 ± 2.15	7.76 ± 1.25	21.6 ± 4.10	0.57 ± 0.20	>300*
V067	IC_{50} ± SEM	0.16 ± 0.09	0.03 ± 0.003	54.2 ± 22.7	0.05 ± 0.01	2.47 ± 0.25	43.5 ± 2.40	0.01 ± 0.01	142.8 ± 8.82
	IC_{90} ± SEM	0.16 ± 0.10	0.05 ± 0.004	>820*	0.92 ± 0.09	4.42 ± 0.22	82.9 ± 8.19	0.14 ± 0.10	>300*
V206	IC_{50} ± SEM	0.52 ± 0.21	0.05 ± 0.007	106.8 ± 15.9	0.25 ± 0.04	2.42 ± 0.34	58.3 ± 0.88	0.06 ± 0.02	196.3 ± 8.82
	IC_{90} ± SEM	0.82 ± 0.44	0.10 ± 0.02	>820*	2.72 ± 0.90	9.04 ± 0.47	70.0 ± 0.58	>0.36*	>300*
V413	IC_{50} ± SEM	1.08 ± 0.32	0.026 ± 0.003	> 820*	0.66 ± 1.38	6.30 ± 1.31	70.0 ± 6.64	0.21 ± 0.21	156.4 ± 25.2
	IC_{90} ± SEM	1.34 ± 0.37	0.033 ± 0.006	> 820*	1.65 ± 4.65	12.1 ± 4.49	96.5 ± 21.1	>1.4*	>300*
V597	IC_{50} ± SEM	0.35 ± 0.21	0.051 ± 0.0003	190.5 ± 36.7	0.35 ± 0.03	3.40 ± 0.32	55.6 ± 2.03	0.07 ± 0.03	181.6 ± 17.6
	IC_{90} ± SEM	0.45 ± 0.22	0.054 ± 0.001	>820*	2.74 ± 0.48	8.67 ± 1.19	64.3 ± 0133	>0.36*	>300*
V631	IC_{50} ± SEM	0.40 ± 0.16	0.08 ± 0.003	172.7 ± 80.9	0.38 ± 0.21	2.73 ± 0.09	59.2 ± 2.33	0.11 ± 0.05	148.5 ± 15.3
	IC_{90} ± SEM	0.52 ± 0.29	0.16 ± 0.04	>820*	6.68 ± 3.08	10.0 ± 1.33	67.0 ± 4.84	>0.36*	>300*
Davis	IC_{50} ± SEM	0.35 ± 0.12	0.03 ± 0.003	> 820*	1.03 ± 0.62	5.08 ± 1.08	100.3 ± 16.7	0.21 ± 0.11	106.6 ± 3.33
	IC_{90} ± SEM	0.41 ± 0.18	0.05 ± 0.005	> 820*	4.12 ± 0.87	8.73 ± 0.62	>120*	>1.4*	>300*
HB1	IC_{50} ± SEM	0.50 ± 0.40	0.04 ± 0.006	7.64 ± 0.87	0.01 ± 0.001	1.58 ± 0.15	113.2 ± 3.33	0.006 ± 0.006	85.9 ± 14.1
	IC_{90} ± SEM	0.55 ± 0.46	0.41 ± 0.27	12.0 ± 3.10	0.02 ± 0.003	1.90 ± 0.06	>120*	0.008 ± 0.012	>300*
HB4	IC_{50} ± SEM	0.61 ± 0.48	0.07 ± 0.048	> 820*	4.51 ± 0.30	6.25 ± 0.53	72.0 ± 7.27	0.96 ± 0.16	128.9 ± 37.2
	IC_{90} ± SEM	0.90 ± 1.16	0.70 ± 1.01	> 820*	11.7 ± 1.53	8.92 ± 0.64	97.7 ± 22.6	4.96 ± 2.97	>300*
Ty	IC_{50} ± SEM	0.73 ± 0.30	0.12 ± 0.049	126.7 ± 52.3	1.50 ± 1.10	7.60 ± 1.80	68.3 ± 18.8	0.56 ± 0.42	112.7 ± 8.82
	IC_{90} ± SEM	0.88 ± 0.48	2.31 ± 0.92	>820*	4.42 ± 2.15	13.5 ± 1.33	>120*	4.35 ± 2.06	>300*

* $IC_{50/90}$ values exceeded the maximum concentration of drug tested for this clinical isolate

TABLE S2 Data from post hoc comparisons of calculated Growth Rates among the 11 isolates using Tukey-Kramer's Multiple Comparisons test. The mean difference is determined from the calculated average growth rate of 3 biological replicates per isolate.

Comparisons (A vs. B)	Mean Difference (A-B)	95% Confidence Interval of Difference	Significance	Multiplicity Adjusted P-value
Nf69 vs. V597	-9.3	-15.11 to -3.490	***	0.0004
V067 vs. V597	-8.2	-14.01 to -2.390	**	0.0019
V206 vs. V413	5.9	0.08962 to 11.71	*	0.0445
V413 vs. V597	-11.1	-16.91 to -5.290	****	<0.0001
V413 vs. V631	-6.1	-11.91 to -0.2896	*	0.0343
V597 vs. 6088	5.9	0.08962 to 11.71	*	0.0445
V597 vs. Davis	7.2	1.390 to 13.01	**	0.0076
V597 vs. HB1	10.6	4.790 to 16.41	****	<0.0001
V597 vs. HB4	7.9	2.090 to 13.71	**	0.0028
V597 vs. Ty	6.7	0.8896 to 12.51	*	0.0152

'*' = Significant ($0.01 < p < 0.05$), '**' = Very significant ($0.001 < p < 0.01$), '***' = Extremely significant ($0.0001 < p < 0.001$), '****' = Extremely significant ($p < 0.0001$).

References

1. Rice CA, Colon BL, Alp M, Göker H, Boykin DW, Kyle DE. 2015. Bis-benzimidazole hits against Naegleria fowleri discovered with new high-throughput screens. Antimicrobial Agents and Chemotherapy 59:2037-2044.
